# Analysis of the *CCR5* gene coding region diversity in five South American populations reveals two new non-synonymous alleles in Amerindians and high CCR5*D32 frequency in Euro-Brazilians

**DOI:** 10.1590/S1415-47572009005000011

**Published:** 2009-01-16

**Authors:** Angelica B. W. Boldt, Lodércio Culpi, Luiza T. Tsuneto, Ilíada R. Souza, Jürgen F. J. Kun, Maria Luiza Petzl-Erler

**Affiliations:** 1 Laboratório de Genética Molecular Humana, Departamento de Genética, Universidade Federal do Paraná, Curitiba, PRBrazil; 2 Laboratório de Polimorfismos e Ligação, Departamento de Genética, Universidade Federal do Paraná, Curitiba, PRBrazil; 3 Department of Human Parasitology, Institute of Tropical Medicine, University of Tübingen, TübingenGermany; 4 Universidade Estadual de Maringá, Maringá, PRBrazil; 5 Universidade Federal de Santa Catarina, Florianópolis, SCBrazil

**Keywords:** * CCR5*, Brazilian, Amerindian, HIV, polymorphism

## Abstract

The CC chemokine receptor 5 (CCR5) molecule is an important co-receptor for HIV. The effect of the CCR5*D32 allele in susceptibility to HIV infection and AIDS disease is well known. Other alleles than CCR5*D32 have not been analysed before, neither in Amerindians nor in the majority of the populations all over the world. We investigated the distribution of the *CCR5* coding region alleles in South Brazil and noticed a high CCR5*D32 frequency in the Euro-Brazilian population of the Paraná State (9.3%), which is the highest thus far reported for Latin America. The *D32* frequency is even higher among the Euro-Brazilian Mennonites (14.2%). This allele is uncommon in Afro-Brazilians (2.0%), rare in the Guarani Amerindians (0.4%) and absent in the Kaingang Amerindians and the Oriental-Brazilians. *R223Q* is common in the Oriental-Brazilians (7.7%) and *R60S* in the Afro-Brazilians (5.0%). *A29S* and *L55Q* present an impaired response to β-chemokines and occurred in Afro- and Euro-Brazilians with cumulative frequencies of 4.4% and 2.7%, respectively. Two new non-synonymous alleles were found in Amerindians: *C323F* (*g.3729G* > *T*) in Guarani (1.4%) and *Y68C* (*g.2964A* > *G*) in Kaingang (10.3%). The functional characteristics of these alleles should be defined and considered in epidemiological investigations about HIV-1 infection and AIDS incidence in Amerindian populations.

## Introduction

The human immunodeficiency virus type 1 (HIV-1) epidemic shows great variation among the different Brazilian regions. A progressive reduction in the number of deaths from acquired immunodeficiency syndrome (AIDS) was observed after the introduction of potent antiretroviral therapy in 1996, but the deceleration of the AIDS epidemic was not homogenous throughout all the Brazilian regions (Brito *et al.*, 2005). The Southeast region has experienced the lowest increase in the AIDS epidemic from 1990 to 1996, contrasting with a steep rise in the North and South regions (Szwarcwald *et al.*, 2000). Since 1996, the incidence rates of AIDS in Brazil as a whole and in the State of São Paulo in particular show a trend towards stability, whereas in the Brazilian Northeast the incidence rates of the disease continue to grow (Brito *et al.*, 2005). The different spreading of the disease is due to multiple variables, including biological, behavioural, demographic and economic/political factors that influence the rate of contact between infected and susceptible individuals, as well as the individual's infectiousness and susceptibility. Among these factors are genetic variants of host genes that facilitate or hamper viral entry into the cells and modulate immune responses against the infection.

The chemokine (C-C motif) receptor 5 gene (*CCR5*) comprises three exons. The polypeptide of 352 amino acid residues is encoded by exon 3 (formerly named exon 4) (Mummidi *et al.*, 1997). CCR5 transduces the signals of several different chemokines in phagocytes and T lymphocytes and serves as an essential co-receptor for the entry of R5-tropic HIV-1 into those cells (Blanpain *et al.*, 2001). This is the viral form that most frequently infects people in Brazil (Ferraro *et al.*, 2001). Therefore, *CCR5* alleles that code for proteins poorly or not expressed at the cell surface are strong candidates for protection against the infection and for the delay of AIDS onset. This is the case of the truncated CCR5*D32 allele, and probably also of the *Fs299* and *R60S* alleles (Dean *et al.*, 1996; Shioda *et al.*, 2001; Tamasauskas *et al.*, 2001). CCR5*D32 was also favourably associated with autoimmune diseases such as multiple sclerosis, rheumatoid arthritis and type 1 diabetes mellitus, but increases the risk for abdominal aortic aneurysm and sarcoidosis (for a review, see Navratilova, 2006).

The interaction between the CCR5 receptor and its ligands can block HIV-1 entry and thus retard disease progression. The *A29S* and *L55Q* alleles encode products with a reduced affinity for (C-C motif) chemokines and might be associated with a shorter time interval from HIV infection to AIDS onset (Howard *et al.*, 1999).

During AIDS, the acquisition of mutations in the HIV-1 gp120 envelope glycoprotein gene leads to the switch from primary R5 (CCR5-using) to highly cytopathic X4 (CXCR4-using) HIV-1 variants. According to the somatic hypermutation hypothesis, this switch takes place in the germinal center B cells, due to aberrant somatic hypermutation of the gp-120-coding region of the HIV-1 env gene (Suslov, 2004). This process seems to be more effective in CCR5*D32 heterozygotes, which were found at a 2.5 times higher risk of harbouring X4 HIV-1 variants before the onset of highly active antiretroviral therapy. The presence of X4 variants in the patients seems not to compromise the therapy outcome (Brumme *et al.*, 2005), whereas the presence of a CCR5*D32 allele was found associated with a better response (Accetturi *et al.*, 2000; Guerin *et al.*, 2000).

In order to better understand the diversity of the *CCR*5 gene and to supply data for studies on the functional effect and epidemiological consequences of the *CCR5* variants, we investigated the distribution of CCR5*D32 and other known exon 3 coding region *CCR5* alleles in five populations of South Brazil. These alleles and their known functional characteristics are listed in Table 1. We also sequenced part of the coding region of the gene, in order to search for new variants.

## Materials and Methods

### Samples

One hundred and seventy two Afro-Brazilians, 172 Euro-Brazilians, 18 Oriental-Brazilians, 115 Guarani (89 of which belong to the M'byá sub-group) and 160 Kaingang were investigated. All individuals were randomly selected and live in the State of Paraná, in South Brazil, with the exception of 26 Guarani belonging to the Kaiowá and Ñandeva subgroups which live in the State of Mato Grosso do Sul in Central-Western Brazil. For some *CCR5* alleles, the number of individuals analysed was lower. The classification of individuals as Euro-Brazilians and Afro-Brazilians was based on morphological features. The Euro-Brazilians included 53 unrelated German-speaking individuals whose ancestors came from or joined Mennonite settlements in South Brazil. No HLA genotyping data was available for this subsample. Based on HLA allelic frequencies previously determined for all other population samples, an average European component of 34% and an average Amerindian component of 6% were estimated for the Afro-Brazilians. For the non-Mennonite Euro-Brazilians, the African and Amerindian components are approximately 9% and 5%, respectively (Braun-Prado *et al.*, 2000; Probst CM, MSc Dissertation, Universidade Federal do Paraná, Curitiba, 2000; Probst *et al.*, 2000). The average admixture values of the Guarani and Kaingang with the immigrants from Europe and Africa were estimated to be 4% and 7%, respectively (Petzl-Erler *et al.*, 1993; Probst CM, MSc Dissertation, Universidade Federal do Paraná, Curitiba, 2000; Tsuneto *et al.*, 2003). The gene flow between these two Amerindian groups is also low, being approximately 1.4% in Guarani and 0.5% in Kaingang (Petzl-Erler *et al.*, 1993).

### Typing method

DNA was extracted from peripheral blood cells using the standard phenol/chloroform/isoamyl alcohol or salting-out techniques. The coding region of exon 3 of the *CCR5* gene was amplified by PCR as described previously (Boldt and Petzl-Erler, 2002). The product was applied on nylon membranes in the form of dot-blots and allowed to hybridize with sequence-specific oligonucleotide probes (SSOP, Table 2), according to the protocol of the XII International Histocompatibility Workshop (Fernandez-Viña and Bignon, 1997). Part of the coding region of exon 3 was additionally sequenced using the CCR5rev internal primer in 13 Guarani and 29 Kaingang, one Euro-Brazilian and five Oriental-Brazilian samples. These samples and 59 additional Guarani and 55 additional Kaingang samples were also sequenced using the CCR5for internal primer. One Guarani M'bya individual was genotyped only by sequencing. Sequencing reactions were performed with BigDye Terminator version 1.1 chemistry (Applied Biosystems, Foster City, CA). The sequences of the primers and probes are listed in Table 2.

### Statistical analysis

Genotype and allele frequencies were obtained by direct counting with the aid of the Convert program version 1.1 (Program distributed by the author, CM Probst). The Hardy-Weinberg equilibrium and population homogeneity hypotheses were tested using the approach of Guo and Thompson and the Raymond and Rousset test, respectively, in the ARLEQUIN software package version 3.1 (http://cmpg.unibe.ch/software/arlequin3) (Excoffier *et al.*, 2005). p = 0.05 was adopted as the significance limit.

## Results

The *CCR5* genotype distributions met the Hardy-Weinberg equilibrium expectations in all populations. The frequency of the most common *CCR5* allele varied from 88% to 100% (Table 3). Alleles *Fs299* and *P332P* were not observed in the population samples studied. The other alleles were seen in at least one population, at frequencies varying from about 0.5% to 5% for most of them, except *D32* and *R223Q*.

Three *D32* homozygotes were found among the Euro-Brazilians. The *D32* heterozygote frequencies were 4.1% (7/172) in Afro-, 15.1% (26/172) in Euro-Brazilians, and 0.9% (1/115) in the Guarani Amerindians. We did not find the CCR5*D32 allele in Oriental-Brazilians nor in the Kaingang Amerindians. This allele was more frequent in the Euro-Brazilian sample (9.3%) than in any other sample previously investigated in Latin America (Table 4). The frequency of the *D32* allele rose to 14.2% in a subsample of 53 German-speaking Euro-Brazilians, whose ancestors came from or joined Mennonite settlements in the past. Two of the three homozygotes and 15 of the 26 heterozygotes seen in the Euro-Brazilian sample belonged to this group. Nevertheless, there was no statistically significant difference between the frequency distribution of the *CCR5* genotypes of the Mennonite and the non-Mennonite Euro-Brazilians investigated (p = 0.08, exact test of population differentiation).

Allele *R223Q* was observed in Oriental-Brazilians but not in the other population samples (Table 3). It occurred in the heterozygotic state in two of 13 Oriental-Brazilians (heterozygote frequency of 15.4%).

Sequencing analysis of the coding region of exon 3 revealed a new allele in the Guarani (*g.3729G* > *T*), causing the substitution of cysteine by phenylalanine at amino acid residue 323 (*p.Cys323Phe*) in the C-terminal intracellular segment of the protein. The *p.Cys323Phe* allele occurred in two heterozygotes out of the 72 Guarani individuals whose DNA was sequenced, which allowed estimating an allelic frequency of 1.4% in the Guarani population. The DNA carrying this variant was reamplified and resequenced to confirm the presence of this new allele. In the Kaingang, sequencing revealed another new allele (*g.2964A* > *G*) causing the substitution of tyrosine by cysteine at the conserved residue 68 (*p.Tyr68Cys*) in the second transmembrane part of the protein. This allele occurred in five heterozygotes out of 29 sequenced individuals, which allowed estimating a frequency of 10.3% in the Kaingang population. We also confirmed the presence of the *R223Q* allele in one heterozygote out of the 5 Oriental-Brazilians whose exon 3 was sequenced.

## Discussion

This is the first study investigating the *A29S* and *R60S* alleles in European-derived populations. Also, alleles other than *D32* have not been analysed before in Amerindians. Based on the *CCR5* allelic frequencies in the Chinese, North- and South-American populations (Table 3), it is possible to infer that *A29S,R60S,A335V* and *Y339F* most likely originated in Africa; *L55Q* and *D32* in Europe; *R223Q* and *Fs299* in Asia. The *P332P* allele, found only once in one heterozygote Afro-American (Ansari-Lari *et al.*, 1997), was not found in our population samples nor in screenings of about 700 Afro-Americans, 700 Euro-Americans and 785 Chinese (Ansari-Lari *et al.*, 1997; Carrington *et al.*, 1997; Zhao *et al.*, 2005). The presence of the *D32* allele in the Guarani seems to be the result of gene flow from Neo-Brazilians, as suggested for Mura and Kaingang in another study (Hunemeier *et al.*, 2005).

The high *D32* frequency in Euro-Brazilians is similar to the frequencies found in Central Europe (Stephens *et al.*, 1998). It is compatible with the greater European component in the Euro-Brazilian population of the Paraná State, in comparison to other, previously analysed Brazilian populations of predominantly European ancestry (Probst *et al.*, 2000). The *D32* frequency in the Mennonite subsample is two times higher than in the non-Mennonite Euro-Brazilian subsample and equals the high *D32* frequencies in North Europe (Stephens *et al.*, 1998; Yudin *et al.*, 1998). The frequency of *L55Q*, another allele with likely European origin, is three times higher in Mennonite compared to non-Mennonite Euro-Brazilians. The Mennonites have Friesian origin (North of Germany and the Netherlands) and exist as a religious Anabaptist group since the second half of the XVI century. The majority of individuals in this subsample are direct descendants from 200 Mennonite families that left their villages in the Ukraine and in Siberia and arrived in South Brazil in 1930 (Pauls Jr., 1976). Thus, a founder or bottleneck effect associated to random genetic drift most probably caused the rise in the *D32* and *L55Q* allelic frequencies in this population.

The *R223Q* allele is the most frequent variant in the Chinese population. It is equally distributed in HIV-1 infected and non-infected Chinese groups and has similar HIV-1 co-receptor activity as the major *CCR5* allele (Zhao *et al.*, 2005). Other populations have thus far not been investigated. We also found this allele among the Oriental-Brazilians.

The cysteine residue we found mutated to phenylalanine at codon 323 (*p.Cys323Phe*) in two heterozygote Guarani individuals is not conserved in CCR2, the homologous C-C chemokine-receptor protein with the highest sequence similarity to CCR5 (75%). The substitution of the same residue by alanine was found to decrease the expression of the CCR5 protein on the cellular membrane by preventing receptor palmitoylation (Blanpain *et al.*, 2001). A change in the secondary structure and function may also be expected from the replacement of this residue by phenylalanine. In the Kaingang, sequencing revealed another new allele (*g.2964A* > *G*) causing the substitution of tyrosine by cysteine at the otherwise conserved residue 68 (*p.Tyr68Cys*) in the second transmembrane part of the protein. This allele seems to be very common in the Kaingang population and restricted to it. Possible protective effects of both alleles regarding HIV-1 infection and progression to AIDS have to be established in appropriate cohorts attending Amerindian(-derived) populations.

In summary, we studied the distribution of the CCR5 coding region alleles in various Brazilian populations and noticed a high *D32* frequency in the Euro-Brazilian population of the Paraná State in South Brazil. The *D32* frequency is even higher among the Mennonites and is the highest thus far reported for Latin America. We also identified two new coding *CCR5* mutations in the Amerindian populations, whose functional characteristics should be defined and considered in epidemiological investigations about HIV-1 infection and AIDS incidence in Amerindian populations.

## Figures and Tables

**Table 1:** Functional characteristics (*in vitro*) of previously known *CCR5* alleles*.

DNA level	g.2846G > T	g.2925T > A	g.2941G > T	g.3315_3346del32	g.3429G > A	g.3654delC	g.3757C > T	g.3765C > T	g.3777A > T
SNP (NCBI) database	rs1800939	rs1799863	rs1800940	-	rs1800452	rs34962689	-	rs1800944	rs1800945
Protein level	*p.Ala29Ser*	*p.Leu55Gln*	*p.Arg60Ser*	*p.Ser79_Thr88del*	*p.Arg223Gln*	*p.Phe299fs*	silent	*p.Ala335Val*	*p.Tyr339Phe*
Common nomenclature	*A29S*	*L55Q*	*R60S*	*D32*	*R223Q*	*Fs299*	*P332P*	*A335V*	*Y339F*
Gene transcription							?		
Membrane expression			X	X		X			
HIV-1 infection			X	X		X			
Response to chemokines	x	x		X		X			

*(Blanpain *et al.*, 2000; Howard *et al.*, 1999; Zhao *et al.*, 2005) x: impaired/ diminished; X: blocked or absent; ?: unknown. The allele nomenclature at the DNA and protein levels follows guidelines of the Human Genome Variation Society (http://www.genomic.unimelb.edu.au/mdi/mutnomen/). For practical purposes, we used the common nomenclature adopted by most authors throughout this article (Ansari-Lari *et al.*, 1997; Carrington *et al.*, 1997; Zhao *et al.*, 2005). “Major allele” DNA and protein reference sequences were AF031237 and NP_000570, respectively. Numbers for alleles nominated at the DNA and protein levels obey the nucleotide and amino acid residue numbers in the reference sequences.

**Table 2:** *CCR5* PCR primers and sequence-specific probes.

	Sequence 5'→ 3'	Variant
PCR primer CCR5m	TATGCACAGGGTGGAACAAG	——————————-
PCR primer CCR5jn	CACAACTCTGACTGGGTCAC	——————————-
Seq. primer CCR5for	AATGAGAAGAAGAGGCACAGGGCT	——————————-
Probe CCR5 9- CCR5 9+	AAGCAAATC**G**CAGCC AAGCAAATC**T**CAGCC	*+* *A29S*
Probe CCR5 1- CCR5 1+	CTCATCC**T**GATAAAC CTCATCC**A**GATAAAC	*+* *L55Q*
Probe CCR5 10- CCR5 10+	GCAAAAG**G**CTGAAGA GCAAAAG**T**CTGAAGA	*+* *R60S*
Probe CCR5 2- CCR5 2+	**CAGTATCAATTCTGG** CCATACATTAAAGATAG	+ *D32*
Probe CCR5 3+ CCR5 3-	CTCTGTTTC**G**GTGTC CTCTGCTTC**A**GTGTC	+ *R223Q*
Probe CCR5 14- CCR5 14+	CATCTATG**CC**TTTGT TCATCTATG**C**TTTGT	+ *Fs299*
Probe CCR5 6- CCR5 6+	AGGCTCC**C**GAGCGAG AGGCTCC**T**GAGCGAG	+ *P332P*
Probe CCR5 7- CCR5 7+	GAGCGAG**C**AAGCTCA GAGCGAG**T**AAGCTCA	+ *A335V*
Probe CCR5 8- CCR5 8+	TCAGTTT**A**CACCCGA TCAGTTT**T**CACCCGA	+ *Y339F*

PCR: polymerase chain reaction Seq.: sequencing; +: major allele; in bold: variant nucleotides.

**Table 3:** *CCR5* allelic frequencies and standard deviations in various populations.

Population	*A29S*	*L55Q*	*R60S*	*D32*	*R223Q*	*Fs299*	*P332P*	*A335V*	*Y339F*
Afro-American^1,2^ n = 50	0.015 ± 0.015 (32)	0.008 ± 0.003 (332)	0.013 ± 0.013 (38)	0.019 ± 0.003 (1015)	0	0	0.01 ± 0.01	0.025 ± 0.007 (242)	0.026 ± 0.015 (58)
*Afro-Brazilian* n = 37	0.017 ± 0.017 (29)	0.027 ± 0.019	0.05 ± 0.05 (10)	0.02 ± 0.008 (172)	0	0	0	0.015 ± 0.007 (172)	0 (11)
Euro-American^1,2^ n = 50	nt	0.041 ± 0.008 (354)	nt	0.1 ± 0.004 (2605)	0.016 ± 0.016 (32)	0	0	0.006 ± 0.006 (87)	0 (121)
*Euro-Brazilian* n = 172	0.007 ± 0.007 (69)	0.02 ± 0.008	0 (156)	0.093 ± 0.016	0	0	0	0.003 ± 0.003	0.006 ± 0.004
*Mennonites* n = 53	0 (30)	0.038 ± 0.019	0 (39)	0.142 ± 0.034	0	0	0	0	0
*Non-Mennonites* n = 119	0.013 ± 0.013 (39)	0.013 ± 0.007	0 (117)	0.071 ± 0.017	0	0	0	0.004 ± 0.004	0.008 ± 0.006
Chinese^3^ n = 785	0	0	0	0	0.047 ± 0.005	0.005 ± 0.002	0	0	0
Oriental-American^1,2^ n = 100	nt	0	nt	0	0.04 ± 0.014	0.04 ± 0.014	0	0	0
*Oriental-Brazilian* n = 13	nt	0	0 (11)	0 (16)	0.077 ± 0.053	0 (12)	0 (12)	0 (18)	0 (12)
*Guarani* n = 27	0	0 (34)	0	0.004 ± 0.004 (115)	0 (34)	0	0	0.013 ± 0.008 (115)	0
*Kaingang* n = 31	0	0	0	0 (160)	0	0	0	0 (160)	0
Hispanic^1^ n = 50	nt	0.01 ± 0.01	nt	0.03 ± 0.012	0.02 ± 0.014	0	0	0	0

n: number of individuals; nt: not tested; in parenthesis: number of individuals if different from “n”. ^1^(Ansari-Lari *et al.*, 1997); ^2^(Carrington *et al.*, 1997); ^3^(Zhao *et al.*, 2005); shadowed in italics: this work.

**Table 4:** *D32* allelic frequencies and standard deviations in Latin American populations.

Population	n	*D32* freq.	Region	Reference
Admixed Mexican	212	0.014 ± 0.006	————, MX	(Zuniga *et al.*, 2003)
Amerindian Mayo	70	0		
Amerindian Teenek	61	0		
Amerindian Mazatecan	61	0.016 ± 0.011		
Afro-Jamaican	242	0.01 ± 0.005	————, JM	(Hisada *et al.*, 2002)
Colombian	150	0.027 ± 0.009	Medellin, CO	(Diaz *et al.*, 2000)
Amerindian	172	0.009 ± 0.005	Arequipa, PE	(Calzada *et al.*, 2001)
Amerindian Tikuna	191	0	Northwest Amazonas, BR	(Leboute *et al.*, 1999)
Amerindian Baniwa	46	0		
Amerindian Kashinawa	29	0	Southwest Amazonas, BR	(Leboute *et al.*, 1999)
Amerindian Kanamari	34	0		
Amerindian Tiriyó	180	0	North Amazonas, BR	(Grimaldi *et al.*, 2002)
Amerindian Waiampi	221	0		
Six Amerindian groups	89	0	North Pará, BR	(De Pinho Lott Carvalhaes *et al.*, 2004)
Brazilian	394	0.03 ± 0.006		
Afro-Brazilian	67	0.008 ± 0.008		
Oriental-Brazilian	111	0		
Brazilian	104	0.02 ± 0.01	Recife, Pernambuco, BR	(de Souza *et al.*, 2006)
Admixed Brazilian	549	0.026 ± 0.005	Northeast Bahia, BR	(Grimaldi *et al.*, 2002)
Afro-Brazilian	54	0.019 ± 0.013	Rio de Janeiro, BR	(Chies and Hutz, 2003)
Brazilian	115	0.056 ± 0.015	São Paulo, BR	(Munerato *et al.*, 2003)
Brazilian	100	0.035 ± 0.013	Ribeirão Preto, São Paulo, BR	(Passos Jr and Picanço, 1998)
Euro-Brazilian	102	0.044 ± 0.014	Paraná, Santa Catarina and Rio Grande do Sul, BR	(Chies and Hutz, 2003)
Brazilian	127	0.055 ± 0.014		(Kaimen-Maciel *et al.*, 2007)
Eight Amerindian groups	241	0.013 ± 0.005		(Hunemeier *et al.*, 2005)
Afro-Brazilian	172	0.02 ± 0.008	Paraná, BR	
Euro-Brazilian	172	0.093 ± 0.016		
Oriental-Brazilian	16	0		This work.
Guarani	114	0.004 ± 0.004		
Kaingang	160	0		
Brazilian	100	0.05 ± 0.015	Londrina, Paraná, BR	(Brajão de Oliveira *et al.*, 2007)
Euro-Brazilian	99	0.066 ± 0.018	Santa Catarina, BR	(Grimaldi *et al.*, 2002)
Euro-Brazilian	59	0.068 ± 0.023	Alegrete, Rio Grande do Sul, BR	(Vargas *et al.*, 2006)
Afro-Brazilian	13	0.038 ± 0.038		
Admixed Brazilian	31	0.064 ± 0.032		
Afro-Brazilians	58	0.009 ± 0.009	Rio Grande do Sul, BR	(Chies and Hutz, 2003)
Amerindian Chiriguano	42	0.012 ± 0.012	Northwest Argentina, AR	(Mangano *et al.*, 2001)
Argentinean	751	0.03 ± 0.004	————, AR	(Gonzalez *et al.*, 2001)
Chilean	62	0.024 ± 0.014	————, CL	(Desgranges *et al.*, 2001)

n: number of individuals; freq.: frequency; ISO 3166-1 codes indicate countries.
